# Caseload midwifery in a rural Australian setting: A qualitative descriptive study

**DOI:** 10.18332/ejm/131240

**Published:** 2021-01-19

**Authors:** Alia Kashani, Jessica L. Ingberg, Ingegerd Hildingsson

**Affiliations:** 1 Department of Women’s and Children’s Health, Uppsala University, Uppsala, Sweden; 2 Department of Nursing, Mid Sweden University, Sundsvall, Sweden

**Keywords:** midwifery, caseload, continuity of care, on-call service

## Abstract

**INTRODUCTION:**

Midwifery-led continuity of care models are beneficial to women and babies, but might be challenging for midwives. Several studies have, however, shown that midwives report higher job satisfaction and less burnout when working with caseload. Another challenge is to staff such models in rural areas. The aim of this study was to describe midwives’ experiences of working in a caseload model in a rural region of Australia.

**METHODS:**

A qualitative descriptive approach using interviews and thematic analysis was undertaken with eleven midwives.

**RESULTS:**

The overarching theme, ‘A modified caseload model of care in rural Australia creates opportunities for increased job satisfaction despite the challenges involved with being on call’, comprised: two themes, ‘Increased job satisfaction’ and ‘Challenges’; one core theme, ‘Being on call’; and several subthemes. Working with caseload creates job satisfaction and increases vitality and positive feelings about being a midwife. The main difficulty, as well as the necessity with this model, is the challenging aspect of being on call.

**CONCLUSIONS:**

Caseload midwifery builds partnership between the woman and her midwife, it allows flexible working hours and increased autonomy, even when the work affects the midwife’s social life. Being on call allows the midwife to work on the whole scope of midwifery practice and is a basis for the continuity model of care; however, being on call also represents a challenge to be overcome in order to make caseload work. Continuity models may be a means to attract midwives to work in rural areas.

## INTRODUCTION

In recent years, there has been a tendency for midwives to leave the profession worldwide, mainly due to a stressful work environment, extreme workload, and lack of professional recognition^1^. A contemporary meta-ethnographic review of 11 qualitative studies also showed that many midwives considered leaving midwifery and questioned their professional career^2^.

On the other hand, research has also shown that working in continuity models of midwifery care seem to create a healthier work environment for midwives and creates lower levels of burnout, compared to shift work in hospital^3-5^. Following women through pregnancy and childbirth, and thereby building trustful relationships, increases midwives’ feelings of responsibility, accountability, and autonomy, resulting in an overall increase in job satisfaction^6^. Flexibility of working hours and using the full scope of midwifery practice have also been acknowledged. It has also been described that high satisfaction and the feeling of autonomy in the profession gained by working with caseload outweigh the difficulties in working long hours and being on call^7^.

According to the World Health Organization, midwifery-led continuity of care models for pregnant women are beneficial and recommended^8^. Such models involve a known and trusted midwife or a small group of midwives who provide care for women throughout the antenatal, intrapartum and postnatal periods^9^. Continuity models of midwifery care promote women’s and children’s health, and result in better labour and birth outcomes and costs of care^9^.

One important aspect in continuity models is being on call, which is a prerequisite for actually providing continuity, and continuity models could therefore be more or less attractive to midwives, depending on their life situations. Staffing midwifery continuity models could be a challenge, especially in rural areas, and could limit the amount of continuity with a known midwife^9,10^. Caseload models are mainly provided in metropolitan areas^11^. In Australia there are some exceptions, e.g. the midwifery group practice in a rural town in northeast Victoria, Australia, where a midwifery group practice has been running for more than 20 years, suggesting that this is a sustainable and satisfying way of providing maternity services^12,13^.

In Australia, pregnant women have the option of choosing between several models of maternity care depending on whether they have private or public healthcare insurance^14,15^. The major maternity models are provided by either an obstetrician, a general practitioner specialized in obstetric care, or by a team of midwives who collaborate with other professions^16,17^. Continuity models, such as caseload or midwifery group practice, are recommended by the health authorities, but the availability of such models differ. In a study from 2016, Dawson et al.^11^ surveyed a national sample of maternity managers of public hospitals. In all, 31% reported that their hospital offered caseload models, but only 8% of women (mainly of low obstetric risk) had access to continuity models^11^.

### Problem area

High-level evidence demonstrates the benefit of midwifery-led continuity of care models, when compared with standard care models of care, and are beneficial to women, babies, and midwives. There is, however, limited knowledge about midwives’ long-term experiences of working in caseload models, especially in rural areas. The aim was, therefore, to describe midwives’ experiences of and views about working in a caseload midwifery model in a rural setting in Australia.

## METHODS

### Design

A qualitative descriptive interview design was chosen for this study^18^. The procedure started with field observations followed by interviews^19^. The interviews were performed by two midwifery students from Sweden, during their final semester. They had a previous Bachelor’s degree in Nursing (3 years), which is compulsory before applying to Swedish midwifery education (1.5 years) and they had been working as nurses 4–6 years prior to the midwifery education, mainly in surgical clinics and emergency departments. Their previous clinical experience in midwifery was seven weeks of antenatal care in community settings and seven weeks in a labour ward in a large teaching hospital in Sweden with around 4200 annual births. As they were not familiar with the Australian context of care, they ‘shadowed’ the Midwifery Group Practice (MGP) midwives for two weeks during antenatal consultations and on the labour ward. The reason behind this was that the authors are not Australia based and had limited knowledge about the system as there were not, until recently, any continuity models in Sweden.

### Setting

The study was undertaken in a sub-regional health service in northeast Victoria, Australia. The catchment area serves around 100000 people and provides publicly funded maternity care^13^. The level-4 hospital, with a tertiary centre within 3 hours drive, has around 600 births a year and the care is delivered either as shared care (GP/public antenatal clinic, consultant obstetricians) and Midwifery Group Practice. The Midwifery Group Practice (MGP) was implemented in the mid 1990s and has undergone several changes since then. When it was first developed as a pilot program, four midwives offered antenatal, intrapartum and postnatal care for up to six weeks postpartum, for about 80 women per year, regardless of medical risk. Each midwife was allocated and responsible for her own caseload, but the women had the opportunity to meet all midwives working in the program at least once before giving birth^12,13^. The model was modified in 2002, with five midwives working part-time (50%) and providing care for 100 women per year. Antenatal care was provided in a small house close to the hospital where there was also a lactations clinic and a service for perinatal mental health. The midwives worked in a 1 in 4 rotating on-call schedule and covered each other’s annual leave^13^. From 2008 the number of women increased, and each midwife was allocated a caseload of 30 women and the team leader 20 women. Some women were at this stage excluded from the model due to regulations promoting some women to receive care in secondary or tertiary levels. The model was further challenged in 2017 due to shortage of staff, which meant that fewer women had access to the model. Today, the program is run by five midwives, working 75% of ful-ltime and offers a modified caseload model of care. Each midwife has a caseload of 20–30 women a year and works in partnership with another midwife. The on-call hours have changed from 24 to 12 and they work one weekend in every fifth week. After 12 hours, the ward midwives take over the care. The proportion of midwives working in the MGP was nearly 10% of the hospital-based midwives and 130 women (18.5%) were booked in the MGP in 2017^13^.

### Procedure

The first contact was made with a researcher at the University of Melbourne, who was also employed at the hospital under study and was known to the authors. She forwarded information about the study to the midwives currently working on the caseload model and asked if they were willing to participate in the interview study. After consenting, the midwives in the MGP invited their present and former colleagues, and organized a time and place for the interviews.

### Participants

Inclusion criteria were qualified midwives with a minimum of one year of work experience and presently or formerly working in a caseload model. Each participant was individually interviewed in a private room on-site at the hospital. All data were processed confidentially, and written consent was given prior to interviewing. Ethical approval was granted by the regional ethical committee prior to data collection.

The participating midwives were between 24 and 61 years old; they had 3–39 years of work experience and had been working with caseload from 6 weeks to 23 years. Of the 11 midwives interviewed, five were currently working in the MGP modified caseload model, and six had formerly worked in the MGP and were now performing shift work at the labour/maternity ward, domiciliary care, teaching, or staffing.

### Data collection

The interviews followed a semi-structured interview guide. The questions were developed before the interviews started and mainly based on information found in international literature about midwives’ experiences of working in continuity models^3,4^. The questions were tested in a pilot interview, which was also included in the results. The first question consisted of background information, and the remaining questions covered experiences of working with the model. A set of questions then addressed the on-call aspect of the model. The reason for this was the debate about caseload being a healthy work form or not^3,4,20^.

### Data analysis

All interviews were audio-recorded and transcribed verbatim. Data were analysed through Braun and Clarke’s^21^ six phases of thematic analysis using an inductive, data-driven approach. In the first step, the interviews were listened to several times before transcription to gain an understanding of the content and to become familiar with the data. The transcribed interviews were thereafter printed out and read several times as ideas of meaning started to emerge. Thereafter, initial codes were generated manually through the entire data set and collated into meaningful groups and patterns. When coding of the entire text was done, the work of sorting them into relevant themes started. The themes were developed and processed through inductive method, where the generated themes were explored and compared without having a predetermined coding frame. After identifying preliminary themes, a thematic map was created to identify relationships between the themes. The coding and preliminary themes were then discussed and refined. After refinement, a final thematic map with an overarching theme, two main themes, one core theme and several subthemes was developed. During the process of analysis, all aspects were discussed within the research group and confirmed by the Australian researcher.

### Trustworthiness

Trustworthiness aims to evaluate validity and reliability. In qualitative research, the terms dependability, credibility, transferability and conformability are used to investigate trustworthiness^22^.

#### Dependability

Dependability aims to evaluate the stability and consistency of the data. The data/interviews were personally collected, transcribed verbatim and analysed by the authors. Themes and subthemes were constantly compared and discussed within the research group. In the analysis, the raw data were checked for consistency.

#### Credibility

Credibility is the evaluation of how reliable the data are and how they are constructed and the importance of using a well-established research method^23^. Field observations were made during two weeks; the co-authors ‘shadowed’ the caseload midwives and continuously discussed the model to facilitate understanding of how the caseload model is organized. This was necessary as the co-authors were not at all familiar with the health system in Australia, being citizens of another country with a different health system. The immersion of this fieldwork might have affected the result. However, the interview guide was followed during the interviews, and the questions asked in the same order, which also made it possible to control what was discussed. Clarifying questions were sometimes posed based on the fieldwork and the interviews, but during the analysis all authors applied a bracketing technique for their pre-understanding in order not to colour the emerging themes and categories.

#### Transferability

Transferability is the possibility of using the findings of the research in similar situations or contexts. The method, procedure and setting are thoroughly described, which makes it possible for other researchers to redo this study, thus strengthening transferability. The contextual information (organization, geographical areas, workload restrictions, etc.) is important for comparing circumstances in other countries or in previous research.

#### Conformability

Conformability is the evaluation of the neutrality of the findings. The conformability of the present study was enhanced during the steps of analysis; the data and analysis were provided step-by-step, as suggested by Braun and Clark^21^.

## RESULTS

**Figure 1:**
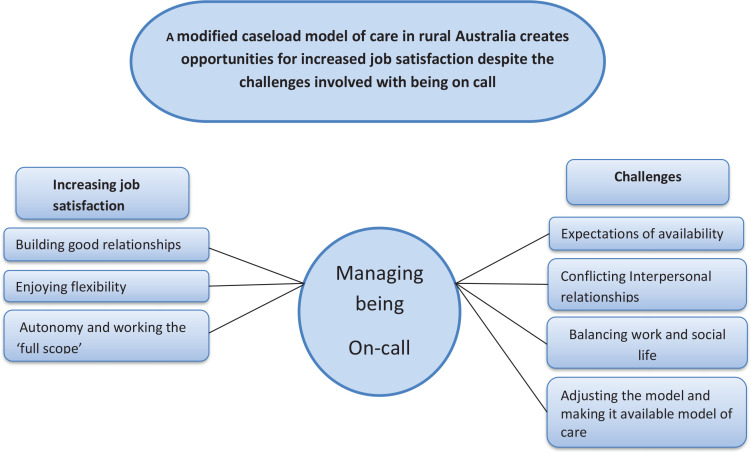
The overarching theme, connected through the core theme of ‘Being on call’, along with the developed themes and subthemes

‘A modified caseload model of care in rural Australia creates opportunities for increased job satisfaction despite the challenges involved’ was the overarching theme in this study. Furthermore, ‘Increased job satisfaction’ and ‘Challenges’ evolved as subthemes, connected through a core theme of ‘Managing being on call’. Forming good relationships with the pregnant women, job satisfaction, flexibility and autonomy were mentioned several times by the midwives as important and positive aspects of working with the model. The model generated positive impacts on the midwife’s private life, vitality and feelings about being a midwife. Different challenges were also raised regarding the caseload, such as the impractical aspects of being on call, which could affect the midwife’s social and family life. Figure 1 shows the themes and subthemes and how they are connected to the core theme of ‘Managing being on call’.

### Increasing job satisfaction

Through the caseload model, midwives had the opportunity to work more flexibly and autonomously than in standard care, which in turn increased job satisfaction. The midwives felt they could offer a positive birth experience because trust had already been established between themselves and the pregnant women during antenatal appointments, and this increased their ability to offer quality care. Providing continuity of care and being available to the women during birth gave each midwife a sense of satisfaction and happiness, in addition to a sense of working the ‘full scope’ of midwifery. Building their confidence and skills in this model was also reported as highly satisfying. All of the participating midwives would recommend other midwives to work with caseload, and several expressed their general feelings about working in caseload:

‘*Yes, I love it (smiling). I just feel like that's what every midwife should be doing, but I know it's not practical for every midwife to be doing caseload. I recommend it, but it depends on the midwife and what she wants out of her midwifery. Some people are very practical and just want to do the skills, others more focused on that social health and advocating women's rights and supporting rights in healthcare and that's something I'm super passionate about, hence being a midwife. So I think caseload, yeah it depends on what you want out of your midwifery career, but yeah I love it so* … (laughing)’ [Midwife 1]

Other midwives also commented:

‘… *this has been the best job I ever had as a midwife for that reason, so you get friendships and you also see the mothers and they are just so fantastic*.’ [Midwife 6]

‘… *definitely feel a lot more job satisfaction. So a lot more satisfied when you're caring for someone you know*.’ [Midwife 3]

#### Building good relationships

Many of the midwives shared the same opinion about the importance of having good relationships not only with the women and their families but also with colleagues and other staff members. Establishing good relationships with the pregnant women could benefit the midwives in several ways, including increasing their vitality and positive feelings about being a midwife as well as benefitting their private lives.

Rewarding outcomes included trust and mutual respect. When trust was established, the midwife felt listened to and that her advice was taken seriously. Having a mutually beneficial relationship also meant getting to know each other on a personal level, which led to ‘good laughs’, feeling more enthusiastic, and a positive working environment. One example follows:

‘*It's definitely positive to get to know our women and their families. It's at a much more personal level, kind of like a friendship in a way, and it's providing a lot more than just care in regard to listening to a baby's heart rate and blood pressure. It's much more personal in a sense of making sure their mental wellbeing is looked after* …’ [Midwife 4]

Another midwife responded:

‘… *but the relationships with the families and the women are just fantastic. So it makes me feel like, as a midwife, I'm contributing to a family*.’ [Midwife 7]

#### Enjoying flexibility

A positive aspect of being on call was the practical flexibility, which led to more efficient work hours and availability. Flexible work hours led to working fewer night and weekend shifts, thereby giving more normal routines and increased vitality. Many of the midwives stated that this improved their social lives in different ways. They could spend more time with family and friends compared to doing shift work at the maternity ward. Some examples:

‘*I like it because you have a little bit more flexibility in a sense that I'm making the appointments with my women so it suits them and me as well. So I like the flexibility of being able to drop off my kids at school and picking them up as much as I can, so I think that's a really big positive. It's just that flexibility with your appointments* …’ [Midwife 5]

‘*Well yes, you have the flexibility, I'll give you an example. A presentation with a child at school, a 9 o'clock school assembly, then you can work your appointments around that but definitely you still got the phone, your accessible, you're still available to the women* …’ [Midwife 8]

#### Autonomy and working the ‘full scope’

All the midwives, to some extent, stated that one of the main factors contributing to making them feel like a true midwife was the autonomy. They felt more independent and they could make their own decisions and do all the practical midwifery tasks. By doing so, they gained confidence, self-trust and satisfaction and were empowered to work their ‘full scope’. The midwives working in the caseload model felt less controlled by the doctors compared to working in the maternity ward. Several stated that they were looking for a challenge and wanted to work the whole spectrum of the midwifery profession, as noted below:

‘… *the way I learned, particularly because I did just midwifery and not nursing, I learned the whole scope of your practice, and I always wanted to work with antenatal care and birth and it really confesses that. Not to put this down on ward midwives, but I think this is how midwifery kind of should be* …’ [Midwife 2]

‘… *yeah, definitely feel you work the entire scope which adds to that satisfaction. You can do the whole lot and see it through and see them become mothers and become parents and work towards that with them* …’ [Midwife 3]

‘… *you should be with women from the very beginning all the way through, and I think women deserve to have a known midwife at their birth and through their pregnancies. It is always how I wanted to do it. Also to be able to use all my midwifery skills*.’ [Midwife 2]

### Challenges

Midwives addressed several problems that could occur with this model of care. For example, it could interfere with social events and create difficulties for family life; in addition, collaborating with other healthcare professionals could be demanding. The most challenging aspect of the caseload model, according to all midwives, was being on call. Many shared the same negative as well as positive opinions regarding management around appointments with work and private life. Midwives who formerly worked in the original caseload model, in comparison with those working in the modified model, felt more negative about the caseload due to the heavier workload.

#### Expectations of availability

Many midwives expressed a need to always be available for the women they provided care for; they felt it was expected, and these expectations could be too demanding at times. The word ‘guilt’ was mentioned when a woman did not end up with her known midwife for the birth or if something went wrong in the birthing suite. Several expressed feeling stressed about being available by phone 24/7 (24 hours, 7 days a week). It was also mentioned that if clear and concise information about the modified model was given at an early stage, the women were more likely to feel confident and to trust their midwives. Women would know when something was out of the ordinary and when they needed to contact their assigned midwife for advice. Some examples illustrating this subtheme:

‘… *I think one of the most difficult things is when women become too dependent on you, because you can't be on call 24/7, and the expectation is that you will be. And they're also expecting you to fulfil all their dreams of a beautiful birth, and you have no control over some things that happen*…’ [Midwife 7]

‘… *women really respect the fact that you're on call and only call with saying if they really genuinely need something and they generally don't abuse the fact that you're available* …’ [Midwife 9]

#### Conflicting Interpersonal relationships

The majority of the midwives stated that the relationship between the caseload midwives and the staff on the maternity ward or with the obstetrician could, at times, be strained and cause feelings of distress. Many expressed a lack of support from the midwives at the maternity ward, and they felt that they had to take care of the women during birth without feeling comfortable about requesting help or advice when needed. One of the midwives stated:

‘ … *it's not very well supported by surrounding midwives and doctors that probably just don't know the meanings of the maternity group practice program. It's a program that's probably put down a lot, and it's very difficult because a lot of people say negative things about it, but yet our focus is on our women and their families. The women are definitely well supported and they love it, so that's the main thing*.’ [Midwife 5]

Another midwife completed:

‘*I would somehow like to change, um the relationship that we have with the rest of the midwives and the maternity service. Feel like it's a real disconnect between, you know, the CMP midwives or the caseload midwives and the caseload women are somehow very different* …’ [Midwife 4]

#### Balancing work and social life

In the caseload model, being on call is an integral part of the model and, at times, is seen as both an asset and a limitation. One positive aspect was the flexibility to be able to offer advice by phone instead of going in for an appointment. Several expressed that it was not possible for them to fully commit to activities or family gatherings and events as a consequence of being on call.

They could not travel too far from the hospital in case they had to come in for an appointment or birth. The potential lack of phone reception was also considered a limitation since the midwife needed to be reachable at all times. The midwives explained that, if they were called in for a birth, they would have to reorganize or rebook work-related appointments and social events. Weekends could be particularly difficult since only one midwife was on call. Many stated that their work made it difficult to plan their private life, especially those with young children at home.

One midwife stated:

‘… *other people sort of say it's been tied up to a phone. I never found it was a problem to answer the telephone. The negative aspect is actually having to leave somewhere and then go home and have to organise when the children were younger, making sure there was someone to care for them when you were called away. So that's really a negative thing. Also, if you've been called out and then called out again within a short space of time, it can be tiring* …’ [Midwife 9]

#### Adjusting the model and making it available

The majority of the midwives stated that the program should be expanded and made available to all women, those with low-risk as well as high-risk pregnancies. One example:

‘… *I think the absolute ideal would be offering this sort of model of care to every woman. I think every woman deserves it*. […] *And we obviously still need our core midwifery staff but to think about increasing midwifery-led care and continuity of care as much as we can, which is something that I'm really focused on. […] The women who are higher risk obstetrically are the ones who miss out and I just have a really strong belief that they're the women who probably need it the most* …’ [Midwife 10]

The majority also suggested that all midwives should be offered an opportunity to work in the model or that a rotating schedule between MGP and the midwives at the ward should be offered. In addition, comments emerged about having their own assigned doctor.

‘… *it would be nice to offer ward midwives to try a period of work in the model* …’ [Midwife 11]

Having one doctor assigned to the MGP would make it easier for the midwives to give the same information and advice to the women instead of having different doctors offering different advice. For example, one midwife commented:

‘*We would also really like to have a doctor assigned to us […] that caseload team have one doctor so when need a doctor it always going to be the same advice, because our problem is that we have so many doctors that you can ask every single one and get different information and it makes it really tricky for us to decide what kind of advice to give women*.’ [Midwife 2]

‘… *I think the ideal would just be increasing the capacity for midwifery-led care and continuity across the board* …’ [Midwife 3]

## DISCUSSION

The findings of this study confirm the importance of the relationship between the woman and her midwife and how it can affect the way the midwife practices her profession. Being on call was a prerequisite for working in a caseload model and a core theme connecting job satisfaction and challenges with the model.

Relationships, flexibility and autonomy were strongly associated with increasing job satisfaction, and these aspects were all connected to being on call. The results show that building good relationships and connecting with the woman is an essential and important factor for job satisfaction and the basis for the caseload model. The relationship was mutual as the midwife felt appreciated and listened to, and experienced that the woman trusted her and her skills as a midwife. These findings align with the partnership model^24^, which emphasizes the midwife’s relationship with the childbearing woman. A study from Denmark found that the relationship with the midwives, from the women’s perspective, was regarded as a professional friendship characterized by equality and inclusiveness. The midwife was also regarded as a navigator in ‘stormy weather’^25^, which can be comparable to our findings. The results of the study by Allen et al.^26^ also align with our results. They aimed to explore how women assigned to the caseload model viewed their midwife in comparison with women assigned to standard care. Women in the caseload model described their midwife as more empowering, which, in turn, led them to feel more involved in their own care and, thereby, increased their feelings of being in control during birth^26^. Being there for the women during pregnancy and birth is one of the greatest benefits of working in the model, as shown in several studies^4,7,27^. The results of the present study also show that midwives felt included in and part of the woman’s family by establishing a relationship during antenatal appointments. A shift in power dynamics was another important issue noted in caseload midwifery from being hierarchical to becoming more equal between women and midwives^28^.

The midwife became familiar with the woman’s medical history and her birth preferences, resulting in more time spent with the woman than reading notes. This is in line with a Danish study that found that a very important aspect for the pregnant women is that they don’t need to repeat their medical history every time they arrive at the hospital; moreover, their plans and preferences for birth are already known and respected by their caseload midwife^25^.

Building good relationships were important, not only with the woman and her family but also with colleagues and other healthcare professionals. It was also mentioned in the interviews that relationships with staff at the maternity ward could sometimes be challenging and result in conflicting interpersonal relationships. In a recent qualitative study of caseload midwifery, the importance was stressed of relationships of trust created with medical colleagues through having a named obstetrician for consultation and referral^28^, similar to the suggested improvements by the interviewed midwives in the present study, in adjusting the model.

There were also challenges related to having close relationships, often imbedded in expectations of availability. Occasionally, the midwives felt that some women were too dependent on the assigned midwife, which could result in feelings of guilt when a woman delivered when her midwife was not on call or if something unplanned happened. The restriction in work hours was also a challenge when the midwife had to hand the care over to a ward midwife. Restricting work hours was seen as a drawback from the pregnant woman’s point of view and could cause disappointment when she was not treated by her assigned midwife^25^. Many of the midwives in the present study also stated that, by forming relationships, they became personally invested in the woman, a finding supported by earlier studies^4,27^, as caseload midwives often feel great responsibility for the women they care for and that, at times, this can be overwhelming^4^.

Being on call is a contributing factor in the ability to offer continuity of care, and was a main focus and a core theme in this study. This led to increased work flexibility as well as flexibility in one’s private life. The combination of working flexible hours and autonomy made it easier for the midwife to manage her personal life while at the same time being available for the women in the caseload. Similar findings have been reported in a grounded theory study from Queensland, Australia^29^. The results of the present study indicate that there are several impractical aspects of being on call, but that the positive aspects still outweigh the negative, as there is usually time for recuperation since women birth at different times. Being able to offer continuity of care was considered more important and meaningful; hence, being on call was not seen as a negative aspect of working in the model. In another study from Western Australia, midwives acknowledged that being on call came with a cost and that it affected not only their sleep and energy levels but also their family life^27^.

While caseload is a flexible model, the results showed that being on call could sometimes intervene with personal plans and make family life difficult, a finding that aligns with studies previously conducted in this field^4,7,20,29^. The midwives had to balance work and social life, which could be challenging. On the other hand, flexibility made it possible to attend a social event but still be at work in the sense that the midwife is available by phone if a woman needs help or counselling. This aspect has also been illustrated previously^29,30^.

Working with caseload was considered a way to advance midwives’ professional skills and strengthen their autonomy. All midwives stated that one of many contributing factors making them really feel like a midwife was the opportunity to work autonomously. This allowed for working the full scope of the midwifery profession, which was not experienced in the same sense when working on the ward, where their work was more controlled by other healthcare professionals. Earlier research aligns with these results, stating that positive aspects of the model include increased autonomy, responsibility for the women, accountability and flexibility of work hours, along with being able to apply midwifery skills and knowledge^4^. Sometimes, autonomy could imply challenging consequences, as illustrated in the subtheme of conflicting interpersonal relationships. The caseload model and the on-call schedule were occasionally questioned and not fully understood by the ward midwives. A feeling of ‘us’ and ‘them’ could develop, which could be very disheartening and could affect patient care. Several of the midwives in the present study suggested that midwives from the ward would be welcomed if they chose to try working in the program in order to gain a better understanding of each other’s workload and increase feelings of collegiality in the midwifery profession. By planning team-building activities and including all staff from the two models of care, a sense of trust could be built between healthcare professionals. This was also highlighted in a study conducted in rural Canada^31^, where inter-professional collaboration between midwives and other healthcare providers was found to be complex and, at times, difficult to improve and where midwives experienced a negative perception of their profession from other healthcare professionals such as doctors and nurses. Their findings further suggested that shared care between the midwife and physician is possible once an implementation of adjustments takes place in regard to each profession’s scope of practice^31^.

Our results show that the majority of the midwives suggested that the program should be expanded and made accessible to all women in the region, regardless of risk status, and that the caseload should be an adjustable and available model of care. Studies show that caseload midwifery is both cost-effective and safe, regardless of the woman’s high- or low-risk pregnancy status^11,16^. Studies have also indicated that the model is more accessible in metropolitan areas of Australia; however, in such areas, women with high obstetric risk or complicated pregnancies are typically excluded^9,16,28^. The results indicate, however, that the caseload model is also preferable in rural areas.

The present study demonstrates the importance of the caseload model being adjustable. This was highlighted by the midwives working with the standard model who expressed greater concerns about being on call than the midwives currently working in the modified model. The midwives working in the modified model work 70% positions, with one weekend on call every fifth week. By contrast, the midwives in the former model have a workload of 100% because they are on call for 24 hours at a time. This indicates that the midwives currently working in the caseload model are more content with their work schedule. It is hoped that, by working fewer weekends and being on call for up to 12 hours instead of 24 hours, the risk of burnout will be decreased.

To summarize, being on call is the biggest part of working in the caseload model, and it can be an advantage but also a limitation; midwives cannot perform caseload work unless they are willing and able to be on call. All eleven midwives also suggested other midwives to try working in the model since they considered that it gave autonomy, included working the full scope of the midwifery profession, and limited interpersonal conflicts. This further indicates that the positive aspects outweigh the negative aspects of working with the model. Research conducted at 43 public hospitals in Australia suggests that midwives should be given the opportunity and experience of working with the caseload model, and that this could be used as a recruitment strategy to interest more midwives in working with this model. The caseload model may not be appropriate for all midwives, but by creating an opportunity to experience it, more midwives may be attracted to working with this model of care^32^. One of the important issues to consider regarding rural locations is the distance to hospitals^10,30^. In the present study, the midwives had excellent access to the hospital as their office was situated within the hospital area. Given these conditions, staffing the caseload model did not seem to be a problem and could explain the long-lasting success of the Midwifery Group Practice in northeast Victoria. In other parts of the world, e.g. Sweden, staffing continuity models have been really difficult due to closure of small labour wards, long distances to hospitals and restricted work hours^10^, which limits the availability of evidence-based midwifery models^9^.

### Strengths and limitations

A strength of the study was the willingness of the participants to share their daily work experiences in the MGP model of midwifery care. The variation of midwives working with both an original caseload and a modified caseload was also seen as a strength, allowing a greater picture of different ways of working with the caseload model of care. It might, however, be possible that, for some of the midwives, the long time since they actively worked in the MGP, might have introduced recall biases. On the other hand, nearly all still worked at the hospital and it is likely that they were fully updated about the model. In the interview situations, interactive questions advanced the understanding of the caseload model and ensured that no contradictions appeared. The interviews were listened to several times, and the transcriptions were proofread and compared with the audio-recordings before hard copies of the raw material were printed. All authors reread all the transcriptions multiple times. The six phases of thematic analysis were used to analyse the raw data material to ensure that no data were omitted^21^. The first and second authors performed the interviews. They did not have any previous relationships with the midwives, apart from the encounters during the ‘shadowing’ period. This period was necessary to learn the context and fully understand the caseload work. One limitation of the study might be the size of the study sample from only one setting. Nevertheless, we reached all midwives who currently worked or had ever worked in the group practice, which resulted in rich data. The description of the modified caseload model in rural Australia could be useful for introducing similar models in other regions of the world, as the results are consistent with those of previous studies on the topic, both from the women’s and midwives’ perspectives and the sustainability of more than 20 years practice suggests that this model provides high quality care.

## CONCLUSIONS

Caseload midwifery builds partnership between the woman and her midwife, it allows flexible work hours and increased autonomy, even when the work affects the social life. Being on call allows the midwife to work on the whole scope of midwifery practice and is a basis for the continuity model of care; however, being on call also represents a challenge to be overcome in order to make caseload work. Continuity models may be a means to attract midwives to work in rural areas. This was mirrored in the overarching theme: ‘A modified caseload model of care in rural Australia creates opportunities for increased job satisfaction despite challenges involved with being on call’. Being on call builds self-confidence in the midwifery profession in the sense that it allows the midwife to work on the whole scope of midwifery, from antenatal care though intrapartum and postpartum care. Being on call is a basis for the continuity model of care, but it is also a challenge to overcome in order to make the caseload model work. Working in continuity models would be a means to attract midwives to work in rural settings.
